# BODY COMPOSITION ASSESSED BY DUAL-ENERGY X-RAY ABSORPTIOMETRY ON METABOLIC PROFILE AND CARDIOVASCULAR RISK IN OBESE PATIENTS PRIOR TO BARIATRIC SURGERY

**DOI:** 10.1590/0102-672020230016e1734

**Published:** 2023-05-26

**Authors:** Cristiane Maria Araújo Tavares de SÁ, Maria Goretti Pessoa de Araújo Burgos, Lucio Vilar Rabelo, Cinthia Katiane Martins Calado, Manoel da Cunha Costa, Thiago Coelho de Aguiar Silva, Renata Adrielle Lima Vieira, Poliana Coelho Cabral

**Affiliations:** 1 Universidade Federal de Pernambuco, Postgraduate Program in Surgery – Recife (PE), Brazil;; 2 Universidade Federal de Pernambuco, Nutrition Department – Recife (PE), Brazil;; 3 Universidade de Pernambuco, School of Physical Education – Recife (PE), Brazil.

**Keywords:** Obesity, Bariatric Surgery, Body Composition, Heart Disease Risk Factors, Obesidade, Cirurgia bariátrica, Composição corporal, Fatores de risco de doenças cardíacas

## Abstract

**BACKGROUND:**

Fat, muscle, and bone are endocrine organs capable of affecting the metabolic profile and cardiovascular risk. Relating these components is important to the establishment of early intervention strategies for overweight patients.

**AIMS:**

This study aimed to evaluate the influence of body mass components on the metabolic profile and cardiovascular risk in the preoperative period of bariatric surgery.

**METHODS:**

A cross-sectional study was conducted with patients admitted for bariatric surgery at a university hospital in the city of Recife, Brazil, between 2018 and 2019. Body composition was determined using dual-energy x-ray absorptiometry. Cardiovascular risk was assessed using the Framingham risk score. Data were collected on anthropometric, clinical, and lifestyle characteristics. The lipid profile (total cholesterol, high-density lipoprotein cholesterol, low-density lipoprotein cholesterol, and triglycerides), blood glucose, and vitamin D were determined using the standard methods of the hospital laboratory.

**RESULTS:**

A total of 60 patients were analyzed, 86.7% of whom had comorbidities, 33.3% had moderate/high cardiovascular risk, and 71.4% had vitamin D insufficiency/deficiency. Lower lean body mass (adjusted PR 3.24; 95%CI 1.19–5.77) was independently associated with the severity of obesity. The body mass index and waist circumference were negatively correlated with lean body mass (r=-0.52; p<0.01)/r=-0.36; p<0.01). Lean body mass was negatively correlated with fat mass (r=-0.26; p<0.05), trunk fat (r=-0.29; p<0.05), fasting glucose (r=-0.26; p<0.05), and bone mineral density (r=-0.26; p<0.05). A total of 84.2% of individuals with less trunk fat tended to have low cardiovascular risk (p=0.05). However, physical inactivity (adjusted PR 2.14; 95%CI 1.19–5.54) and the risk of alcohol dependence (adjusted PR 2.41; 95%CI 1.76–4.15) were the only variables independently associated with cardiovascular risk.

**CONCLUSION:**

Obese patients in the preoperative period of bariatric surgery with less trunk fat tended to have low cardiovascular risk. However, the other components of body mass were also not associated with cardiovascular risk.

## RESUMO

**RACIONAL:**

O tecido adiposo, músculos e ossos representam órgãos endócrinos, capazes de interferir no perfil metabólico e risco cardiovascular. Relacionar esses componentes será importante para estratégias de intervenção precoce, em obesos.

**OBJETIVOS:**

Avaliar a influência dos compartimentos da massa corporal, sobre o perfil metabólico e risco cardiovascular, no pré-operatório de cirurgia bariátrica.

**MÉTODOS:**

Estudo transversal, com pacientes admitidos para cirurgia bariátrica, em hospital universitário. A composição corporal foi avaliada pela absortometria radiológica de dupla energia e o risco cardiovascular pelo escore de risco de Framingham. Foram analisadas variáveis antropométricas, clínicas e de estilo de vida. Foi avaliado perfil lipídico (colesterol total, HDL-c, LDL-c, triglicerídeos), glicemia e vitamina D, utilizando a metodologia padrão.

**RESULTADOS:**

Foram analisados 60 pacientes, 86,7% apresentavam comorbidades, 33,3% risco cardiovascular moderado/elevado e 71,4% insuficiência/deficiência de vitamina D. Menor massa corporal magra associou-se à gravidade da obesidade. O índice de massa corpórea e a circunferência da cintura correlacionaram-se negativamente com a massa corporal magra (r=-0,52; p<0,01)/r=-0,36; p<0,01). A massa corporal magra correlacionou-se negativamente com a massa gorda (r=-0,26; p<0,05), gordura troncular (r=-0,29; p<0,05), glicemia de jejum (r=-0,26; p<0,05) e densidade mineral óssea (r=-0,26; p<0,05). Ao todo, 84,2% dos indivíduos com menos gordura troncular apresentaram tendência a baixo risco cardiovascular (p=0,05). Contudo, inatividade física (razão de prevalência ajustada 2,14; IC95% 1,19–5,54) e risco de dependência alcoólica foram as únicas variáveis independentemente associadas ao risco cardiovascular.

**CONCLUSÕES:**

Os pacientes obesos em pré-operatório de cirurgia bariátrica com menos gordura troncular, apresentaram tendência a baixo risco cadiovascular. Contudo, o risco cardiovascular também não foi associado aos demais componentes da massa corporal.

## INTRODUCTION

According to the World Health Organization, obesity is a pandemic that affects around 13% of the world's population and is a risk factor for various chronic conditions, such as cardiovascular disease^40^. Moreover, the increase in the number of bariatric surgeries performed throughout the world has intensified concerns regarding possible long-term effects, including the loss of lean mass, which can have harmful consequences, requiring the adoption of prevention strategies, which should be implemented as early as possible^23,29^.

A reduction in lean mass, which is considered the main metabolically active component of body mass, reduces energy expenditure at rest and can therefore exert an influence on the rate of weight loss following bariatric surgery^26^. Moreover, a protective role is suggested for lean mass, which has been inversely associated with insulin resistance and seems to attenuate the adverse cardiovascular profile in individuals with excess weight^4^. However, the reduction in fat mass is among the effects of bariatric surgery and leads to an improvement in metabolic status that results in reductions in cardiovascular risk (CVR), insulin resistance, and the risk of type 2 diabetes mellitus^26^.

The body mass index (BMI) and waist circumference (WC) are the most widely used measures in the assessment of the risk of cardiovascular disease^7,8^. However, these anthropometric tools do not distinguish fat mass from lean mass, the latter of which is inversely associated with the risk of cardiovascular disease^4,8^.

The measurement of the components of body mass (fat and lean mass) is important for knowledge on nutritional and health status and is a useful predictor of chronic diseases as well as the determination of the prognosis of various disease processes^10^. However, the literature on the prediction of CVR using these parameters in individuals with obesity is scarce^27^. Dual-energy x-ray absorptiometry (DEXA) is considered the gold standard for the determination of body composition^14,34^.

The present study is the first to investigate the influence of the components of body mass obtained using DEXA on the metabolic profile and CVR determined using the Framingham risk score^22^ in patients with obesity scheduled to undergo bariatric surgery.

## METHODS

A cross-sectional study was conducted with adult patients admitted to undergo bariatric surgery at the Oswaldo Cruz University Hospital of Universidade de Pernambuco (UPE) in the city of Recife (PE), Brazil, between 2018 and 2019.

Individuals with a history of psychiatric illness, those on chronic glucocorticoid use, those with physical disabilities, and those admitted for re-surgery due to weight regain were excluded. Patients with weight greater than 158 kg were also excluded due to the maximum capacity of the DEXA equipment.

The anthropometric measures assessed were weight, height, WC, and neck circumference (NC). After the determination of weight and height, the BMI was calculated, and the degree of obesity was classified according to the criteria of the American Society for Metabolic & Bariatric Surgery: grade I obesity: BMI 30–34.9 kg/m^2^; grade II obesity: BMI 35–39.9 kg/m^2^; grade III obesity: BMI 40–49.9 kg/m^2^; grade IV obesity: BMI 50–59.9 kg/m^2^; and grade V obesity: BMI 60 kg/m^21^.

WC was measured with the participant standing and the tape measure positioned approximately 2 cm above the navel due to the difficulty in determining the midpoint between the last rib and iliac crest in patients with severe obesity. The reading was taken in duplicate at the end of expiration.

NC was determined with the participant standing erect, head positioned on the Frankfurt horizontal plane, and gaze directed forward. The tape measure was placed perpendicularly along the axis of the neck at the midpoint from the cervical spine to the mid-anterior portion of the neck. For men with the laryngeal prominence, the measurement was taken below the prominence.

DEXA was used for the determination of body mass components through a full-body scan using the Lunar Prodigy DF+14.319 Radiation^TM^ equipment (Madison, WI, USA)^10^. The following variables were quantified: percentage of lean mass; lean mass in kg; percentage of fat mass; fat mass in kg; percentual of trunk fat; trunk fat in kg; bone mineral density (BMD) (in g/cm^2^), and bone mineral content (in g)^34^.

DEXA is considered the gold standard for the determination of body composition in individuals with obesity due to its precision, accuracy, and safety as well as the direct measurement of lean mass, fat mass (adipose tissue), and bone mass (BMD)^34,14^. This is a relatively fast, noninvasive, safe method with a minimal dose of radiation^34,13^.

For the biochemical analysis, routine laboratory exams were used at the general surgery service of the university hospital. The following were considered altered values: total cholesterol (TC) >190 mg/dL; high-density lipoprotein cholesterol (HDL-c) <40 mg/dL for men and <50 mg/dL for women; low-density lipoprotein cholesterol (LDL-c) >130 mg/dL; and triglycerides (TG) >150 mg/dL, as suggested by the 2017 guidelines of the Brazilian Society of Cardiology^1^. Diabetes mellitus and prediabetes were defined as fasting glucose (FG) of 126 mg/dL and FG between 100 and 125 mg/dL, respectively, according to the criteria of the American Diabetes Association^3^.

Vitamin D status was defined according to the guidelines of the Endocrine Society: vitamin D deficiency: 25OH vitamin D (25OHD) <20 ng/mL; insufficiency: 25OHD between 20 and 29.9 ng/mL; and sufficiency: 25OHD=30 ng/mL^15^.

The investigation of diseases associated with severe obesity was performed through interviews with the patients and by consulting the patient files for the diagnosis recorded by the physician.

The Framingham score was used for the determination of coronary risk. This score enables the calculation of the absolute risk of coronary events in 10 years^20^. The score may be positive (risk factor) or negative (protection factor) for the calculation of the estimate through the use of the Cox regression model and includes age, blood pressure (systolic and diastolic), TC, HDL-c, smoking, and diabetes mellitus^1,13^. After the determination of the score for each variable, the points were summed and the classification was performed considering low risk to be equal to or lower than 9%, moderate risk to be 10 to 19%, and high risk to be equal to or higher than 20%^1,20^.

As a secondary objective, lifestyle characteristics associated with CVR were investigated (smoking, degree of physical activity, and alcohol intake). The short version of the International Physical Activity Questionnaire (IPAQ) was used for the determination of physical activity level^28^. The Alcohol Use Disorders Identification Test (AUDIT) was used for the investigation of alcohol intake^24^.

Statistical analyses were performed with the aid of the Statistical Package for Social Sciences (SPSS, version 13.0, SPSS Inc., Chicago, IL, USA). The Kolmogorov-Smirnov test was used to determine the normality of continuous variables. As Gaussian distribution was demonstrated, the data were expressed as mean and standard deviation. The Student's t-test was used for the pairwise comparison of means. Pearson's correlation coefficients were calculated for the analysis of correlations between the variables of interest, with the creation of an exploratory correlation matrix. Either the chi-square test or Fisher's exact test was used to test associations between dichotomous variables (p<5%).

Unadjusted and adjusted prevalence ratios (PR) and respective 95% confidence intervals (CIs) were estimated for characteristics associated with CVR and degree of obesity. Poisson regression analysis was employed to determine factors associated with the two outcomes. All variables in with a p-value<0.20 in the bivariate analysis were incorporated into the Poisson regression analysis using the stepwise forward procedure.

This study obtained approval from the UPE Human Research Ethics Committee linked to the HUOC/PROCAPE hospital complex (certificate number: 67051817.9.0000.5192).

## RESULTS

A total of 60 patients were assessed in the preoperative period of bariatric surgery, with a predominance of women (78.3%). Age ranged from 22 to 59 years (mean: 38.9±9.7 years). BMI ranged from 36.8 to 66.2 kg/m^2^ (mean: 47.3±7.0 kg/m^2^).

Table 1 displays the degrees of obesity, with grades II and III accounting for 71.7% of the sample. A total of 84.2% of the individuals with less trunk fat had a tendency of low CVR (p=0.05). No statistically significant influence on CVR was found for the anthropometric measures (BMI, WC, and NC) or the percentages of lean mass and fat mass.

**Table 1:** Cardiovascular risk according to degree of obesity, anthropometric variables, and body composition in patients in preoperative period of bariatric surgery.

		Total		Cardiovascular risk				p-value*
				Moderate/high		Low		
		N	%	N	%	N	%	
Degree of obesity								
	II and III	43	71.7	15	34.9	28	65.1	0.685
	IV and V	17	28.3	05	29.4	12	70.6	
WC (cm)								
	<1st tercile (<118.3)	20	33.3	08	40.0	12	60.0	0.439
	≥1st tercile (≥118.3)	40	66.7	12	30.0	28	70.0	
NC (cm)								
	<1st tercile (<39.0)	16	26.7	04	25.0	12	75.0	0.409
	≥1st tercile (≥39.0)	44	73.3	16	36.4	28	63.6	
LM (%)								
	<1st tercile (<41.4)	20	33.3	07	35.0	13	65.0	0.846
	≥1st tercile (≥41.4)	40	66.7	13	32.5	27	67.5	
FM (%)								
	<1st tercile (<43.4)	20	33.3	6	30.0	14	70.0	0.699
	≥1st tercile (≥43.4)	40	66.7	14	35.0	26	65.0	
TF (%)								
	<1st tercile (<45.6)	19	31.7	03	15.8	16	84.2	0.050
	≥1st tercile (≥45.6)	41	68.3	17	41.5	24	58.5	

* Pearson's χ^2^ test. WC: waist circumference; NC: neck circumference; LM (%): percentage of lean mass; FM (%): percentage of fat mass; TF (%): percentage of trunk fat; cm: centimeters.

With regard to CVR, 33.3% of the patients presented moderate-to-high risk, and 86.7% had some comorbidity, the most frequent of which was hypertension. HDL-c values were associated with low CVR in the majority of the sample. Other metabolic variables exerted no influence on CVR (Table 2).

**Table 2:** Cardiovascular risk according to metabolic and clinical variables in patients in preoperative period of bariatric surgery.

		Total		Cardiovascular risk				p-value*
				Moderate/high		Low		
		N	%	N	%	N	%	
Hypertension								
	Yes	32	53.3	10	31.3	22	68.8	0.714
	No	28	46.7	10	35.7	18	64.3	
Diabetes mellitus								
	Yes	21	35.0	07	33.3	14	66.7	1.000
	No	39	65.0	13	33.3	26	66.7	
Pre-diabetes								
	Yes	08	13.3	01	12.5	07	87.5	0.249^a^
	No	52	86.7	19	36.5	33	63.5	
Triglycerides								
	Normal (≤150 mg/dL)	30	62.5	11	61.1	19	63.3	0.878
	High (>150 mg/dL)	18	37.5	7	38.9	11	36.7	
Total cholesterol								
	Normal (≤190 mg/dL)	24	48.0	10	55.6	14	43.8	0.624
	High (>190 mg/dL)	26	52.0	08	44.4	18	56.3	
HDL-cholesterol								
	Normal	34	69.4	09	50.0	06	80.6	0.025^a^
	Low	15	30.6	09	50.0	25	19.4	
LDL-cholesterol								
	Normal (≤130 mg/dL)	35	71.4	11	31.4	24	68.6	0.516^a^
	High (>130 mg/dL)	14	28.6	06	42.9	08	57.1	
Vitamin D								
	Deficiency/insufficiency	34	68.0	12	75.0	22	64.7	0.467
	Sufficiency	16	32.0	4	25.0	12	35.3	

* Pearson's χ^2^ test.

a Fisher's exact test. Pre-diabetes: fasting glucose between 100 and 125 mg/dL; diabetes mellitus: fasting glucose ≥126 mg/dL; hypertension: blood pressure ≥140/90 mmHg; HDL-c: high-density lipoprotein cholesterol; LDL-c: low-density lipoprotein cholesterol; vitamin D deficiency: 25OH-vitamin D 25OHD <20 ng/mL; vitamin D insufficiency: 25OHD 20–29.9 ng/mL; vitamin D sufficiency: 25OHD ≥30 ng/mL.

Table 3 displays the characteristics of the sample (mean and standard deviation) according to CVR. Individuals classified with moderate CVR were older (p=0.001), had higher FG (p=0.043), and had lower HDL-c (p=0.028) compared to those with low risk. However, no associations were found between the increase in CVR and the components of body mass (fat mass, lean mass, and bone mass), BMI, or vitamin D status.

**Table 3:** Characteristics of sample (mean and standard deviation) according to cardiovascular risk in patients in preoperative period of bariatric surgery.

	Total Mean±SD	Cardiovascular risk		p-value*
		Moderate/high Mean±SD	Low Mean±SD	
Age (years)	38.9±9.7	44.5±10.4	36.2±8.0	0.001
BMI (kg/m^2^)	47.3±7.0	46.9±5.4	47.3±7.6	0.866
NC (cm)	40.7±4.7	40.0±6.2	41.0±3.8	0.476
WC (cm)	125.0±14.0	120.8±11.5	126.2±14.0	0.144
BMD (g/cm^2^)	1.13±0.1	1.2±0.1	1.2±0.1	0.376
BMC (g)	2.3±0.4	2.4±0.3	2.2±0.4	0.105
BMD (z-score)	0.03±1.0	0.1±0.9	-0.0±1.1	0.809
LM (kg)	53.9±10.5	53.5±9.2	53.7±11.1	0.943
LM (%)	45.0±9.2	45.4±8.4	45.0±9.7	0.865
LMI (kg/m^2^)	20.4±337	20.4±3.7	20.4±3.9	0.961
FM (kg)	55.8±8.5	54.0±7.5	56.7±9.0	0.220
FM (%)	46.6±8.4	46.0±9.0	47.2±8.3	0.564
TF (kg)	27.1±5.5	25.9±6.1	27.7±5.2	0.246
TF (%)	47.9±5.4	48.9±4.0	47.2±5.9	0.255
FG (mg/dL)	108.9±33.7	120.9±50.7	101.2±16.4	0.043
HbA1c (%)	6.0±1.1	6.3±1.7	5.8±0.5	0.165
TG (mg/dL)	149.8±59.6	145.8±67.2	149.5±56.5	0.837
TC (mg/dL)	192.6±28.4	195.0±29.7	191.6±28.6	0.691
HDL-c (mg/dL)	42.4±8.4	40.5±8.0	46.0±8.6	0.028
LDL-c (mg/dL)	117.6±27.2	118.3±26.8	117.8±28.4	0.949
VLDL-c (mg/dL)	29.3±12.7	27.8±14.8	29.8±11.9	0.621
25OHD (ng/mL)	26.5±6.4	26.7±3.8	27.0±7.1	0.874
Calcium (mg/dL)	9.1±0.8	9.4±0.9	8.9±0.7	0.022

* Student's t-test. SD: standard deviation; BMI: body mass index; NC: neck circumference; WC: waist circumference; BMD (g/cm^2^): bone mineral density; BMC: bone mineral content; BMD (z-score): bone mineral density by z-score; LM (kg): lean mass in kilograms; LM (%): percentage of lean mass; LMI: lean mass index; FM (kg): fat mass in kilograms; FM (%): percentage of fat mass; TF (kg): trunk fat in kilograms; TF (%): percentage of trunk fat; FG: fasting glucose; HbA1c: glycated hemoglobin; TG: triglycerides; TC: total cholesterol; HDL-c: high-density lipoprotein cholesterol; LDL-c: low-density lipoprotein cholesterol; VLDL-c: very low-density lipoprotein cholesterol; 25OHD: 25OH vitamin D.

The correlation analysis revealed positive correlations between BMI and WC (r=0.43; p<0.01), NC (r=0.33; p<0.05), percentage of fat mass (r=0.40; p<0.01), and FG (r=0.35; p<0.05) as well as a negative correlation between BMI and lean mass (r=-0.52; p<0.01). Except for the negative correlation between WC and percentage of lean mass (r=-0.36; p<0.01), the results were similar to those found for NC, with both measures presenting positive correlations with fat mass (kg) (r=0.44; p<0.01 and r=0.32; p<0.05) and trunk fat (kg) (r=0.33; p<0.05 and r=0.26; p<0.05). Lean mass (kg) was inversely correlated with percentage of fat mass (r=-0.26; p<0.05) and percentage of trunk fat (r=0.29; p<0.05). Similar results were found for percentage of lean mass with fat mass (kg) (r=0.29; p<0.05) and percentage of trunk fat (r=0.29; p<0.05), confirming that greater lean mass denotes less fat, especially in the abdominal region in these patients. A negative correlation was also found between percentage of lean mass and FG (r=-0.26; p<0.05).

In terms of lifestyle, 40% were active, no participants were current smokers, and 16.7% were ex-smokers. Most of them (66.7%) did not drink alcohol and remainder of the sample (91.7%) had a low risk of alcohol dependence. A tendency of lower CVR was found among non-drinkers (p=0.053). In contrast, 80% of individuals at risk of alcohol dependence had high CVR (p=0.038). With regard to physical activity, moderate-to-high CVR was significantly greater among sedentary and insufficiently active individuals compared to active individuals (44.4 vs. 16.7%; p=0.025). No significant difference in moderate/high CVR was found between non-smokers and ex-smokers (30 vs. 50%, p=0.278).

Table 4 displays the adjusted PR in the final model obtained by Poisson regression. After controlling for potential confounding factors, the only variables that remained independently associated with CVR were physical inactivity (adjusted PR 2.14; 95%CI 1.19–5.54) and the risk of alcohol dependence determined by the AUDIT (adjusted PR 2.41; 95%CI 1.76–4.15).

**Table 4:** Unadjusted and adjusted prevalence ratios for characteristics associated with cardiovascular risk in patients in preoperative period of bariatric surgery.

	PR_Unadjusted_	95%CI	p-value	PR_Adjusted_	95%CI	p-value
Male sex	1.95	0.98–3.86	0.076	1.24	0.86–2.67	0.096
Sedentary/Insuf. active	2.67	1.01–7.01	0.025	2.14	1.19–5.54	0.012
Alcohol intake	2.00	1.00–4.00	0.053	1.89	0.94–3.34	0.076
Risk – AUDIT	2.75	1.51–5.02	0.038	2.41	1.76–4.15	0.006
Trunk fat ≥1st tercile	2.63	0.87–7.89	0.050	2.33	0.75–5.67	0.078

Poisson regression – adjusted model for cardiovascular risk. CI: confidence interval; PR: prevalence ratio; p-values are obtained from chi-square test; AUDIT: Alcohol Use Disorders Identification Test; Insuf. active: insufficiently active.

## DISCUSSION

Fat in the trunk region of individuals with obesity exerted a directly proportional influence on CVR. Moreover, a negative impact of the degree of obesity on lean mass was found in the present study.

This is one of the first Brazilian studies to associate trunk fat with CVR in patients in the preoperative period of bariatric surgery. An American study involving 586 individuals with acquired immunodeficiency syndrome found that subcutaneous adipose tissue of the trunk measured by magnetic resonance presented a tendency for an increase in CVR determined by the Framingham score^22^.

Localized fat in the abdominal region has been identified as an important risk factor for the development of cardiovascular disease^12,37^. In the studies cited, individuals with less fat mass in the trunk region had a tendency of low CVR, demonstrating the importance of the location of fat as a predictor of risk. In upper-body obesity, the release of non-esterified fatty acids leads to the ectopic accumulation of fat in the liver and muscles, predisposing individuals to dyslipidemia and an increase in insulin resistance^30^.

Studies have recently suggested that NC is a better predictor of CVR than WC due to the greater release of free fatty acids by neck fat in comparison to visceral and trunk fat, especially in individuals with obesity^2,25,32^. In the present study, NC was positively correlated with fat mass and trunk fat, suggesting that this measure is a safe and precise tool in individuals with obesity. NC also has an advantage over WC, as a greater chance of measurement error occurs with the latter^2,25,32^.

Most obese candidates for bariatric surgery have cardiometabolic comorbidities associated with excess weight, as found in the present investigation (86.7% of patients) and other studies, such as those conducted by Castanha et al.^9^ (84.5%), Baratieri et al.^5^ (80.6%), and Kelles et al.^21^ (60.8%). However, such comorbidities undergo important improvements in most patients after bariatric sugery^33^. According to the literature, hypertension is the most prevalent condition^21,38^. In a systematic review of 73 studies involving 19,543 bariatric patients, the prevalence of hypertension, diabetes mellitus, and dyslipidemia was respectively 44, 24, and 44%^38^. In the series by Castanha et al.^9^, the most frequent comorbidities were hypertension (42.4%), sleep apnea (24.1%), diabetes mellitus (18.2%), and dyslipidemia (9.4%). Among the patients in the present study, 53.3% had hypertension, 52% had high TC, 48.3% had hyperglycemia (35% with diabetes mellitus and 13.3% with pre-diabetes), and 37.5% had hypertriglyceridemia. A higher BMI constitutes a greater risk of such comorbidities, as reported by other authors^5,9,21^. For instance, only 21.7% of patients with grades IV and V obesity had normal blood sugar levels, a statistically significant difference compared to those with grades II and III obesity (p=0.048), and the degree of obesity remained independently associated with higher FG after controlling for potential confounding factors.

CVR was also positively correlated with age and glycemia, possibly because these are part of the criteria of the Framingham score, which was used for this assessment.

No association was found between comorbidities and the reduction in lean mass or the increase in percentages of fat mass or trunk fat, but all patients had fat mass and trunk fat values well above the limit of normality and nearly 90% had comorbidities related to obesity. A reduction in the percentage of lean mass was also found with the increase in BMI and, after controlling for confounding factors, the severity of obesity remained independently associated with a lower percentage of lean mass.

When BMI is associated with the loss of lean mass and an increase in fat mass, which are generally linked to a sedentary lifestyle and a nutritionally inadequate diet, individuals can develop what is denominated sarcopenic obesity^19^. This condition was first described in 2008^35^ and is characterized by the deterioration of bones (osteopenia/osteoporosis) and muscles (sarcopenia), as well as excess adipose tissue (overweight/obesity, including the redistribution of fat in the visceral area and adipogenesis in bone and muscle tissues)^11,18,19,35^. As a result, a pro-inflammatory environment develops, with an increase in oxidative stress and insulin resistance, which favors the aggravation of the metabolic profile and an increase in CVR^16^.

In the present study, WC, which is a measure that reflects obesity in the abdominal region, was negatively correlated with the percentage of lean mass, demonstrating the influence of the central distribution of fat on lean mass. This finding is in agreement with data described in previous studies that identified an increased abdominal circumference as a significant risk factor for sarcopenia^16,17,35^. We also found that lean mass was negatively correlated with fat mass and trunk fat, demonstrating the important role of lean mass in the increase in energy expenditure and less accumulation of trunk (abdominal) fat.

The percentage of lean mass seemed to have a protective effect with regard to glycemia, as a negative correlation was found between these variables. Studies have shown that hyperglycemia is the main component of the metabolic syndrome related to sarcopenia, demonstrating the important relationship between mass and peripheral insulin sensitivity^16,17,35^. In the present investigation, however, both fat mass (in kg) and the percentage of mass were negatively correlated with FG, possibly due to the greater need for the use of glucose-lowering medications in individuals with more fat mass. However, the degree of obesity remained independently associated with higher FG values after controlling for potential confounding factors.

With regard to lifestyle, population-based studies have demonstrated that moderate to frequent alcohol intake is related to an increase in total fat mass and abdominal fat as well as an increase in appetite^36^. In the present study, 75% of the patients who did not consume alcohol had low CVR. In contrast, 80% of those considered at risk for being alcohol dependent based on the AUDIT had a moderate-to-high CVR, and this variable remained independently associated with CVR after controlling for potential confounding factors.

Physical activity is another important aspect of lifestyle that has been associated with a reduction in insulin resistance, weight loss, and improvements in cardiometabolic variables and quality of life^31^. A recent study with a sample similar to that of the present investigation showed that a sedentary lifestyle is associated with a greater consumption of carbohydrates^39^. In the present study, only 16.7% of active patients had moderate-to-high CVR. The adjusted analysis revealed that sedentarism/insufficient physical activity remained independently associated with CVR.

Investigating plasma deficiencies of micronutrients in the preoperative period, vitamin D deficiency, and insufficiency were found in 68% of the participants. Some observational studies indicate an association between low vitamin D status and an increased risk of cardiovascular disorders, which was not confirmed in randomized controlled trials^6^. In the present investigation, no correlation was found between serum levels of vitamin D and BMD or CVR.

One of the limitations of the present study was the small sample size due to the availability and logistics of DEXA and the exclusion of some patients who surpassed the width of the surface of the equipment. However, our findings contribute to a better understanding of the relationship between the degree of adiposity, characteristics of the components of body mass, and metabolic health. Moreover, NC can be suggested as a practical, innovative, and effective tool in the assessment of adiposity and the routine follow-up of bariatric patients, considering the positive correlations with fat mass and trunk fat.

## CONCLUSION

Obese patients in the preoperative period of bariatric surgery with less trunk fat had a tendency of low CVR. Physical inactivity and the risk of alcohol dependence were the only variables that remained independently associated with CVR.
